# Characterization of in vivo metabolites in rat urine following an oral dose of masitinib by liquid chromatography tandem mass spectrometry

**DOI:** 10.1186/s13065-018-0429-y

**Published:** 2018-05-15

**Authors:** Adnan A. Kadi, Sawsan M. Amer, Hany W. Darwish, Mohamed W. Attwa

**Affiliations:** 1 Department of Pharmaceutical Chemistry, College of Pharmacy, King Saud University, P.O. Box 2457, Riyadh, 11451 Saudi Arabia; 2 Analytical Chemistry Department, Faculty of Pharmacy, Cairo University, Kasr El-Aini St, Cairo, 11562 Egypt

**Keywords:** Masitinib, In vivo metabolism, Sprague–Dawley rats, Phase II glucuronide conjugates

## Abstract

Masitinib (MST) is an orally administered drug that targets mast cells and macrophages, important cells for immunity, by inhibiting a limited number of tyrosine kinases. It is currently registered in Europe and USA for the treatment of mast cell tumors in dogs. AB Science announced that the European Medicines Agency has accepted a conditional marketing authorization application for MST to treat amyotrophic lateral sclerosis. In our work, we focused on studying in vivo metabolism of MST in Sprague–Dawley rats. Single oral dose of MST (33 mg kg^−1^) was given to Sprague–Dawley rats (kept in metabolic cages) using oral gavage. Urine was collected and filtered at 0, 6, 12, 18, 24, 48, 72 and 96 h from MST dosing. An equal amount of ACN was added to urine samples. Both organic and aqueous layers were injected into liquid chromatography-tandem mass spectrometry (LC–MS/MS) to detect in vivo phase I and phase II MST metabolites. The current work reports the identification and characterization of twenty in vivo phase I and four in vivo phase II metabolites of MST by LC–MS/MS. Phase I metabolic pathways were reduction, demethylation, hydroxylation, oxidative deamination, oxidation and N-oxide formation. Phase II metabolic pathways were the direct conjugation of MST, N-demethyl metabolites and oxidative metabolites with glucuronic acid. Part of MST dose was excreted unchanged in urine. The literature review showed no previous articles have been made on in vivo metabolism of MST or detailed structural identification of the formed in vivo phase I and phase II metabolites.

**Electronic supplementary material:**

The online version of this article (10.1186/s13065-018-0429-y) contains supplementary material, which is available to authorized users.

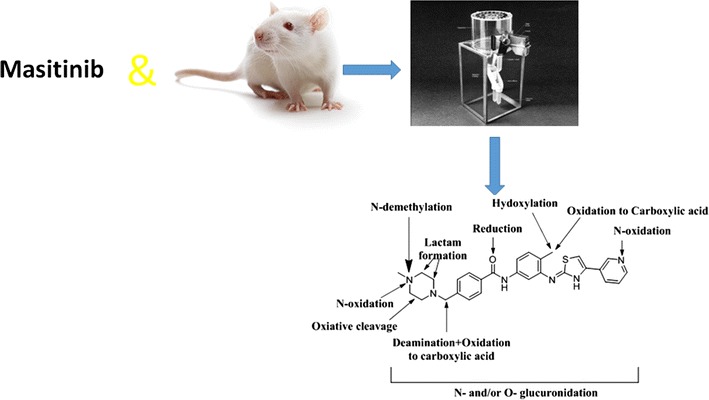

## Introduction

Cancer became a major reason of death [1]. More than four millions new cancer cases reported in developed countries [2, 3]. Molecular targeting strategies were used to treat distributed cancer depending on identifying the tumor suppressors and oncogenes involved in the progress of human cancers [4]. Tyrosine kinase inhibitors (TKIs) (e.g. masitinib) are compounds that target tyrosine kinases enzymes, which are responsible for the activation of numerous proteins in a number of cell signaling pathways. They initiate or stop many functions inside living cells [5]. Blocking the selected activation of these proteins has been shown to have therapeutic benefits in cancer diseases and central nervous system disorders mast cells and macrophages [6, 7]. Tyrosine kinase inhibitors (TKIs) are considered a very important class of targeted therapy [8].

**Fig. 1:**
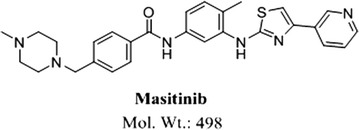
Chemical structure of MST

MST (Fig. 1) is new orally administered TKIs. It is already registered in Europe and USA for the treatment of mast cell tumors in dogs [9]. MST is approved under the trade name masivet in Europe and Kinavet in the USA at a dose of 12.5 mg kg^−1^ per day [10]. Toxicity profile of MST is lower than other TKIs [11]. MST selectively inhibits c-kit tyrosine kinase blocking stem cell factor induced proliferation. It exhibits more activity and selectivity against KIT than imatinib in in vitro studies [11]. In 3 October 2016, AB Science announced that the EMA has accepted a conditional marketing authorization application for MST to treat ALS in human. MST found to be effective for the treatment of severely symptomatic indolent or smouldering systemic mastocytosis [12].

Drug metabolism research is an integral part of the drug discovery process and is very often the factor that determines the success of a given drug to be marketed and clinically used [13]. Drug metabolism research is generally conducted using in vitro and/or in vivo techniques. In vitro techniques involve the incubation of drugs with different types of in vitro preparations (e.g. liver microsomes, hepatocytes) isolated from rats and subsequent sample processing and analysis using spectroscopic techniques [14, 15]. In vivo techniques involve the administration of a single dose of the drug to rat, and the subsequent collection of urine that contain the drugs and their potential metabolites. In this work, we focused in the in vivo phase I metabolites and in vivo phase II MST metabolites identification using LC–MS/MS [16]. All measurements were done using Agilent LC–MS/MS system that consisted of LC (Agilent HPLC 1200) coupled to MS/MS detector (6410 QqQ MS) through an electrospray ionization source (Agilent Technologies, USA) [17].

MST chemical structure contains cyclic tertiary amine. Phase I metabolism of cyclic tertiary amines produces metabolites of oxidative products including N-dealkylation, ring hydroxylation, α-carbonyl formation, N-oxygenation, and ring opening metabolites that can be formed through iminium ion intermediates [18, 19].

## Chemicals and methods

### Chemicals

All chemicals are listed in Table 1.

**Table 1:** List of materials and chemicals

Name^a^	Source
Masitinib	LC Labs (USA)
Tween 80	Eurostar Scientific Ltd. (UK)
Ammonium formate, HPLC grade acetonitrile (ACN), Dimethyl Sulfoxide (DMSO), Polyethylene glycol 300 (PEG 300) and formic acid	Sigma-Aldrich (USA).
Water (HPLC grade)	Milli-Q plus purification system (USA)
Sprague–Dawley rats	Animal Care Center, College of Pharmacy, King Saud University (Saudi Arabia)

^a^ All solvent are HPLC grade and reference powders are of AR grade

### In vivo metabolism of MST in Sprague–Dawley Rats

#### Rat dosing protocol

Male Sprague–Dawley rats (n = 6, average: 340 g, 4 weeks of age) were housed individually in special purpose metabolism cages. Cages are placed in the animal care facility in a 12 h light/dark cycle (7:00–19:00) and were allowed free access to standard animal feed and water that were placed in the special food and water compartments attached to the metabolism cages. Rats were acclimated in metabolism cages for 72 h prior to the start of the study. MST was formulated in (4% DMSO, 30% PEG 300, 5% Tween 80, HPLC H_2_O) for oral dosing of rats. Doses were individually calculated for each rat such that everyone receives a specific dose. The average dose of MST (Kinavet-CA1) in dogs was 10 mg kg^−1^. By using the following equations [20–22]:

$${\text{Rat }}\left( {\frac{{\rm mg}}{{\rm kg}}} \right) = {\text{Dog }}\left( {\frac{{\rm mg}}{{\rm kg}}} \right) * {\text{Km ratio}}$$

$${\text{Rat }}\left( {\frac{\rm{mg}}{\rm{kg}}} \right) = 10*20/6$$

$${\text{Rat }}\left( {\frac{\rm{mg}}{\rm{kg}}} \right) = 200/6$$

$${\text{Rat }}\left( {\frac{\rm{mg}}{\rm{kg}}} \right) = 33.3\left( {\frac{\rm{mg}}{\rm{kg}}} \right)$$

So the dose for each rat was 33.3 mg/kg. All rats except one were given a single dose of MST. All MST doses were administered by oral gavage. Urine draining into the special urine compartments fitted to the metabolism cages were collected prior to drug dosing as blank control reference and at 6, 12, 18, 24, 48, 72 and 96 h following MST dosing. Urine samples taken from all metabolism cages were pooled together, labeled, and stored at (− 20 °C).

#### Sample preparation

Urine samples were thawed to room temperature and filtered over 0.45 µm syringe filters. Liquid liquid extraction (LLC) was used to extract MST and its related metabolites. Equal volume of ice cold acetonitrile (ACN) was added to each sample then vigorously shaken by vortexing for 1 min. Phase separation [23, 24] between an aqueous sample and a water-miscible solvent (ACN) into two layers achieved by using ice cold ACN that was added to urine and the mixture was stored at 4 °C overnight [25]. Low temperature leads to phase separation of ACN/urine mixture. The *p*H of urine and the nature of urine matrix which contains high concentration of salt participated in phase separation [26]. As we did not want to miss any MST-related metabolites, both layers were removed and evaporated to dryness under stream of nitrogen. The dried extracts were reconstituted in 1 mL of mobile phase and transferred to 1.5 mL HPLC vials for LC–MS/MS analysis. Control urine samples obtained from rats prior to drug dosing were prepared in the exact way described for each method of sample purification.

### LC–MS/MS conditions

The LC–MS/MS parameters optimized for chromatographic separation and identification of rat urine extract components are listed in Table 2.

**Table 2:** Adjusted parameters of the supposed LC–MS/MS methodology

Parameters of LC			Parameters of MS/MS	
HPLC	Agilent 1200		Mass spectrometer	Agilent 6410 QQQ
Gradient mobile phase	A: H_2_O (10 mM Ammonium formate, pH:4.1)		Ionization source	Positive ESI
	B: ACN			Drying gas: N_2_ gas Flow rate (12 L/min) Pressure (55 psi)
	Flow rate: 0.2 mL/min			
	Run time: 45 min			
	Injection volume: 20 µL			
Agilent eclipse plus C_18_ column	Length	50 mm		ESI temperature: 350 °C
	Internal diameter	2.1 mm		Capillary voltage: 4000 V
	Particle size	1.8 μm	Collision gas	High purity N_2_
	Temperature:	24 °C	Modes	Mass scan and product ion (PI)
Gradient system	Time	%B	Analyte	MST and its related in vivo phase I and phase II metabolites
	0	5		
	40	40	Mass parameters	Fragmentor voltage: 130 V
	43	40		
	45	5		
	Post time (15 min)	5		Collision energy of 20 eV

### Identification of in vivo MST metabolites

MST-related metabolites were concentrated in the ACN layer while endogenous urine components and polar metabolites (e.g. glucuronide conjugates) were found in the aqueous layer. Extracted ion chromatograms for the expected metabolites were used to find metabolites in the total ion chromatogram of both organic and aqueous layers. PI studies were for the suspected compounds and results were interpreted and compared with the PI of MST. Mass scan and PI scan modes of the triple quadrupole mass analyzer were used for detection of in vivo phase I and phase II MST metabolites. PI mass spectra were used to propose the metabolite chemical structure by reconstructing the marker daughter ions.

## Results and discussion

### Identification of in vivo phase I metabolic pathways of MST

**Fig. 2:**
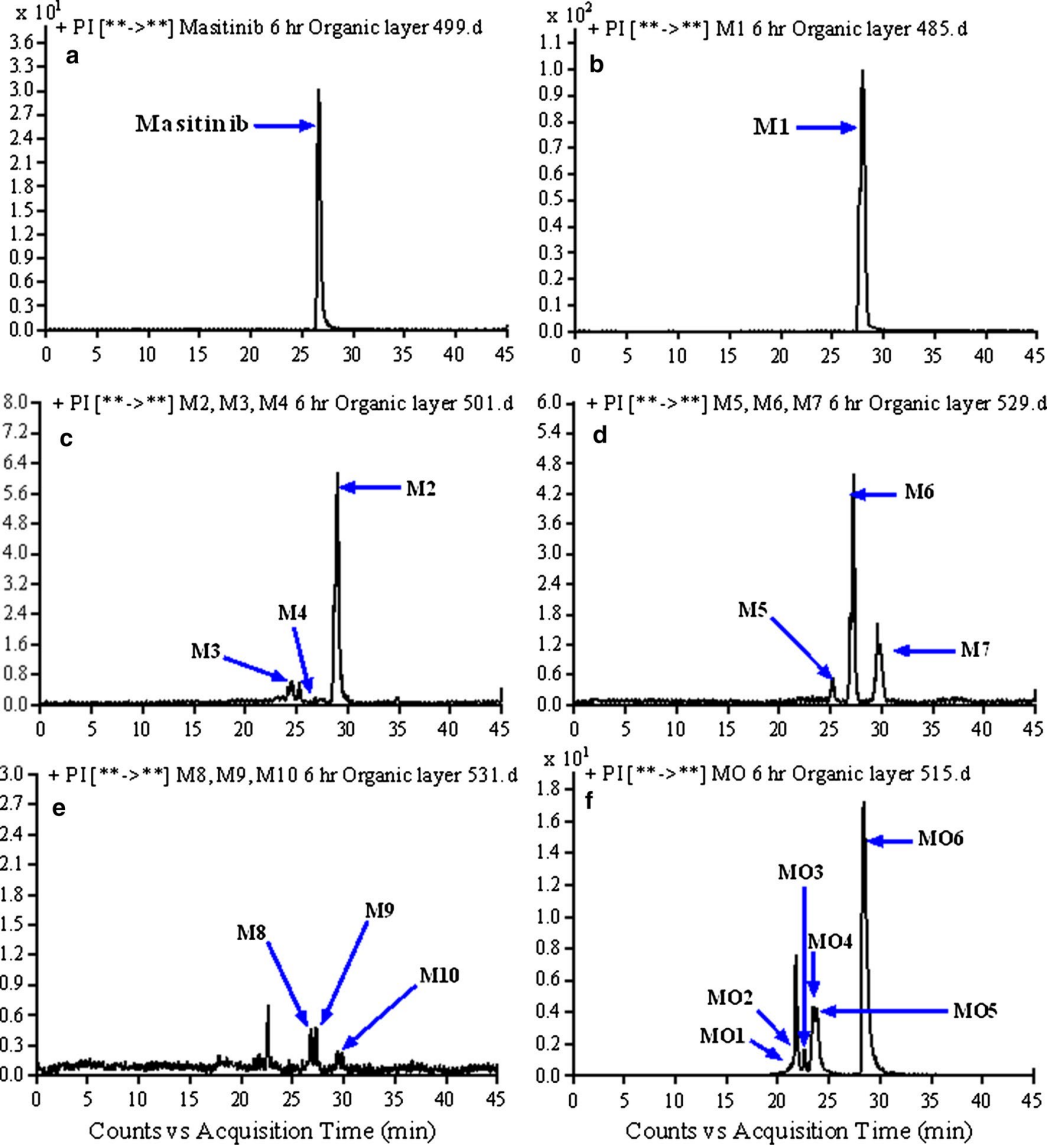
PI chromatograms: **a** (MST), **b** (M1), **c** (M2–M4), **d** (M5–M7), **e** (M8–M10) and **f** (MO1–MO6)

The in vivo metabolites of MST underwent fragmentations similar to that of the parent ion that allowed us to identify and determine changes in the metabolite structures. The product ion mass spectra of some metabolites exhibited particular fragmentation pathways that provided more structural information as shown below. Comparison of PI mass spectra between urine extracts with control samples in addition to the comparison of PI of MST and its anticipated metabolites (Table 3) resulted in the detection of twenty in vivo phase I and four phase II metabolites (Fig. 2). Ten in vivo phase I metabolites are reported in the case of in vitro metabolism [27]. We concentrated on the structural identification of the new ten in vivo phase I and the other four in vivo phase II MST metabolites. Metabolic pathways for in vivo phase I metabolites were supposed to be N-demethylation, N-oxide formation, oxidation, oxidative deamination, reduction, oxidative cleavage, benzyl oxidation and hydroxylation while for phase II metabolites were N-conjugation of MST and the N-demethyl metabolite with glucuronic acid and oxidative metabolites glucuronidation.

**Table 3:** *In vivo* phase I MST metabolites

	[M + H]^+^	PI	RT (min)	In vivo phase I metabolic reaction
MST	499	399	24.9	
M1	485	399	27.9	N-demethylation
M2	501	401	26.6	Carbonyl group reduction
M3	501	400.2, 367.3	24.4	N-demethylation and Hydroxylation of pyridine ring
M4	501	482.9, 399.3	26.5	N-demethylation and Hydroxylation of N-methyl piperazine
M5	529	511, 429	25.1	Benzyl oxidation to carboxylic acid
M6	529	486, 400	26.9	Pyridine ring hydroxylation and N-methyl piperazine oxidation
M7	529	511,482 399, 247	29.6	Oxidation and Hydroxylation of N-methyl piperazine
MO1	515	497.2, 415, 396.8	21.7	N-oxide formation
MO2	515	497.2, 396.9	22.2	Benzylic hydroxylation
MO3	515	497.0, 400.1	23.0	Pyridine ring hydroxylation
MO4	515	497, 399, 415, 217	23.1	Pyridine ring N-oxidation
MO5	515	497, 399, 415, 217	24.0	N-oxidation
MO6	515	428, 415, 400, 381.3, 98.1,	28.0	Piperazine ring N-oxidation
M8	531	488, 402, 123	26.7	Pyridine ring hydroxylation and piperazine ring hydroxylation
M9	531	415, 381, 123	27.3	Piperazine ring hydroxylation and benzyl hydroxylation
M10	531	501, 401	29.3	Oxidative cleavage of N-methyl piperazine ring to carboxylic acid
M11	547	511	30.7	N-oxide formation of pyridine and piperazine ring and Benzylic hydroxylation [27]
MA1	431	255	10.2	Oxidative deamination
MA2	447	271	13.2	Phenyl hydroxylation and oxidative deamination
MA3	447	285, 271, 164, 111	14.5	Benzyl hydroxylation and oxidative deamination

#### MST excretion of in rat urine

**Fig. 3:**
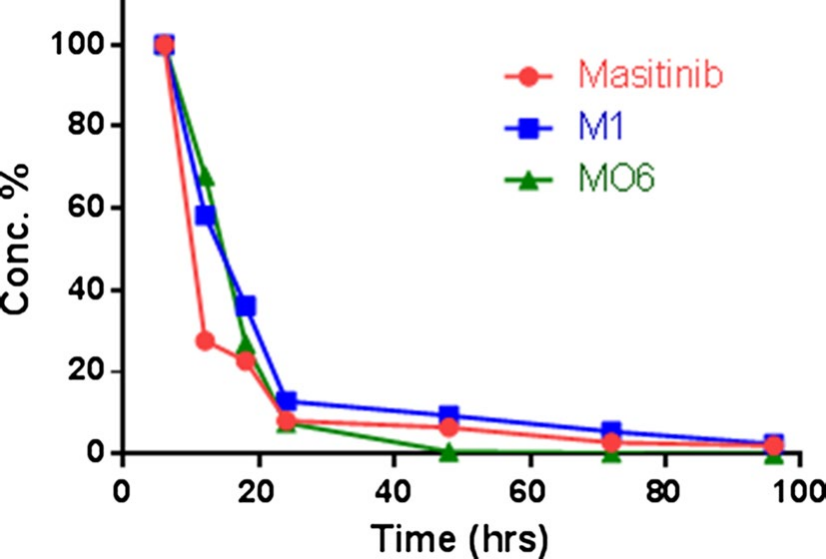
MST, M1 and MO6 excretion rate

Part of the MST oral dose was excreted unmetabolized in rat urine. MST parent ion was detected at *m/z* 499 in full mass scan spectrum. MST of and its major in vivo metabolites (M1 and MO6) excretion in urine was observed after 6 h of dosing. Comparative concentrations of MST, M1 and MO6 were high after 6 h and then began to decline by time until almost vanished after 96 h from dosing as shown in the overlayed PI chromatograms (Check Additional file 1). Peak area ratios of MST and its major metabolite (M1 and MO6) in urine were plotted against time. Peak area ratio of each MST, M1 and MO6 were measured at different collection time considering the biggest peak is 100% (Fig. 3) [28].

**Fig. 4:**
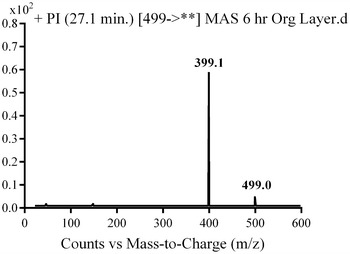
PI of MST parent ion at *m/z* 499

**Scheme 1:**
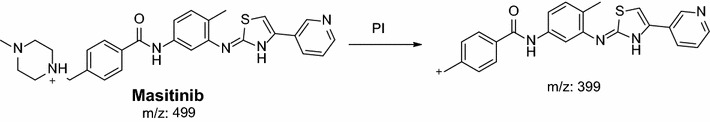
Supposed PI of MST

Fragmentation of MST (Fig. 4) was explained in Scheme 1. Comparison of PI of MST with suspected peaks allowed the identification of metabolic changes in the supposed in vivo metabolites.

#### M1 in vivo phase I metabolite

The major metabolic pathway for MST is N-demethyalation. M1 was detected at *m/z* 485 in mass scan spectrum.

#### M2, M3 and M4 in vivo phase I metabolite

M2, M3 and M4 were detected at *m/z* 501 at different retention times in mass scan spectrum of organic urine extract. PI scan for the three metabolites gave different daughter ions. In the case of M2, parent ion at *m/z* 501 was fragmented to one ion at *m/z* 401. The daughter ion at *m/z* 401 supposed that there is no change in the methyl piperazine group. The metabolic pathway for M2 metabolite was supposed to be the reduction of the carbonyl group.

**Fig. 5:**
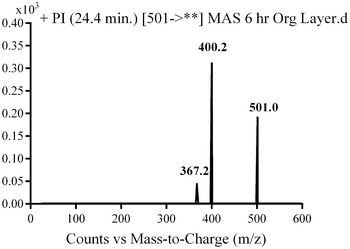
PI mass spectrum of parent ion (M3) at *m/z* 502

**Scheme 2:**
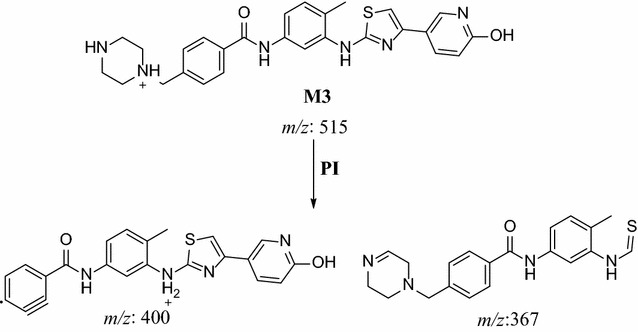
Supposed PIs of M3

In the case of M3, parent ion at *m/z* 501 was fragmented to ions at 400.2 and 367.2 (Fig. 5). Metabolic pathways for M3 were supposed to be hydroxylation of pyridine ring and N-demethylation (Scheme 2).

**Fig. 6:**
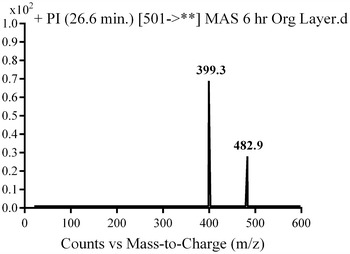
PI mass spectrum of parent ion (M4) at *m/z* 501

**Scheme 3:**
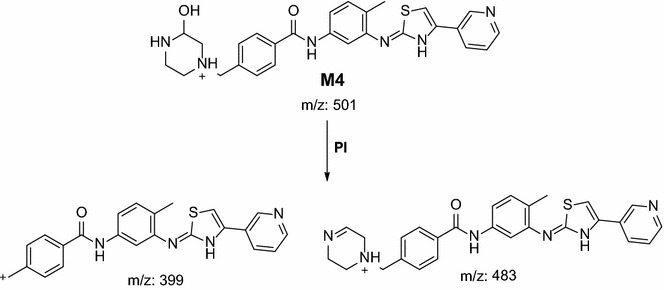
Supposed PIs of M4

In the case of M4, parent ion at *m/z* 501 was fragmented to two daughter ions at *m/z* 483 and at *m/z* 399 (Fig. 6). The daughter ion at *m/z* 399 supposed that there all metabolic changes occured in the methyl piperazine group. Metabolic pathways for M4 metabolite were hydroxylation and N-demethylation of N-methyl piperazine (Scheme 3).

#### MO1 to MO6 in vivo phase I metabolite

Oxidized MST metabolite (M + O) was detected at *m/z* 515 in mass scan spectrum at different retention times. Fragmentation of parent ions at *m/z* 515 gave different daughter ions as shown in the Table 3. The structure of each metabolite was supposed The metabolic pathway for MO metabolites was supposed to be either by hydroxylation or N-oxidation of MST [27].

#### M5, M6 and M7 in vivo phase I metabolite

**Fig. 7:**
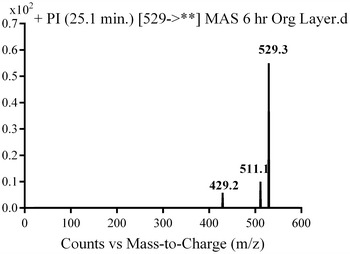
PI mass spectrum of parent ion (M5) at *m/z* 529

**Scheme 4:**
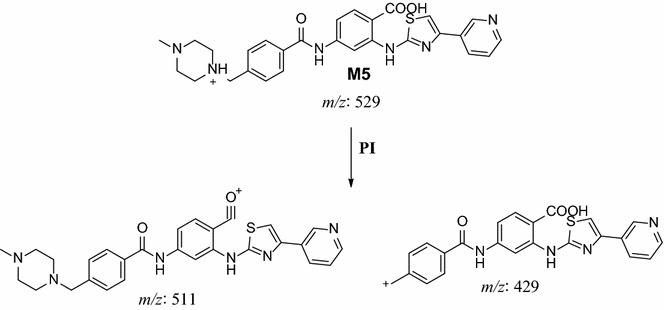
Supposed PIs of M5

M5, M6 and M7 metabolites were detected at *m/z* 529 in full mass scan spectrum at different retention times. PI scan for parent ions at *m/z* 529 gave different daughter ions. In the case of M5, parent ion at *m/z* 529 was fragmented to ions at *m/z* 511 and at *m/z* 429 (Fig. 7). The metabolic pathway for M5 was supposed to be benzyl oxidation to carboxylic acid (Scheme 4).

**Fig. 8:**
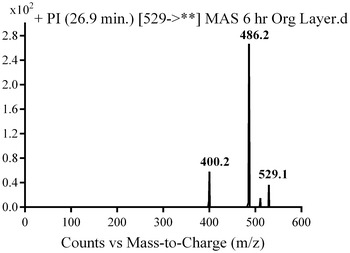
PI mass spectrum of parent ion (M6) at *m/z* 529

**Scheme 5:**
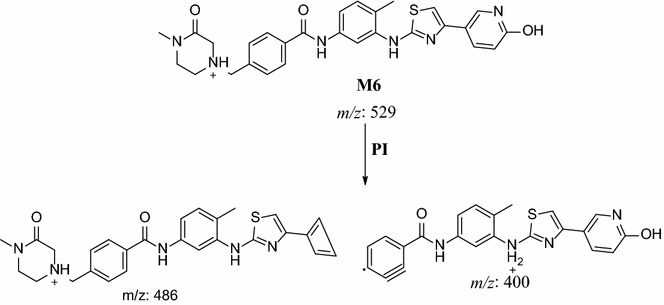
Supposed PIs of M6

In the case of M6, parent ion at *m/z* 529 was fragmented to ions at 486 and 400 (Fig. 8). The metabolic pathway for M6 was supposed to be hydroxylation and oxidation of methyl piperazine ring (Scheme 5).

**Fig. 9:**
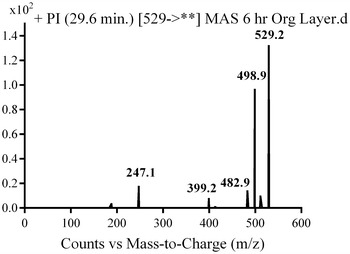
PI mass spectrum of parent ion (M7) at *m/z* 529

**Scheme 6:**
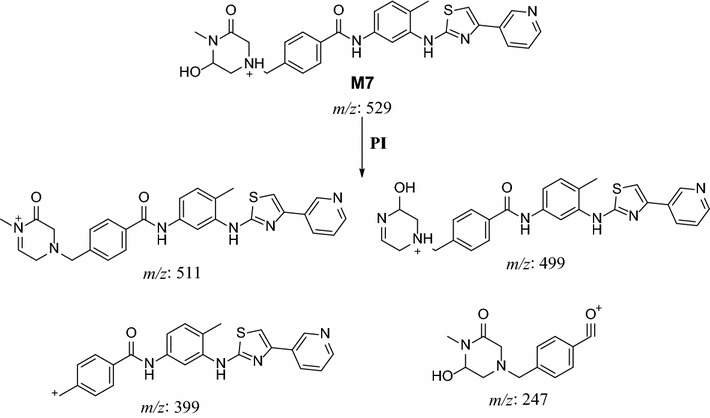
Supposed PIs of M7

In the case of M7, parent ion at *m/z* 529 was fragmented to ions at 511, 399 and 98 (Fig. 9). Metabolic pathways for M7 were supposed to be hydroxylation and oxidation of methyl piperazine ring (Scheme 6).

#### M8, M9 and M10 in vivo phase I metabolite

**Fig. 10:**
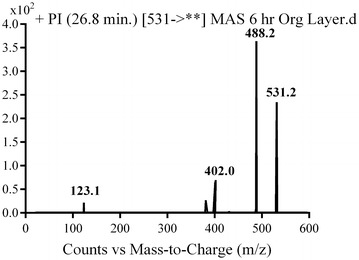
PI mass spectrum of parent ion (M8) at *m/z* 531

**Scheme 7:**
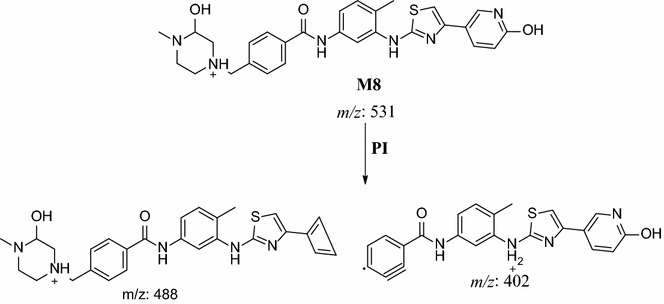
Supposed PIs of M8

M8, M9 and M10 metabolites were detected at *m/z* 531 in full mass scan spectrum at different retention times. PI scan for parent ions at *m/z* 531 gave different daughter ions. In the case of M8, parent ion at *m/z* 531 was fragmented to ions at 488, 402 and 123 (Fig. 10). Metabolic pathways for M8 were supposed to be hydroxylation of pyridine and hydroxylation of methyl piperazine ring (Scheme 7).

**Fig. 11:**
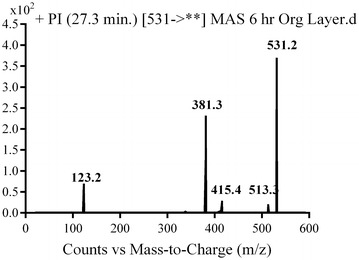
PI mass spectrum of parent ion (M9) at *m/z* 531

**Scheme 8:**
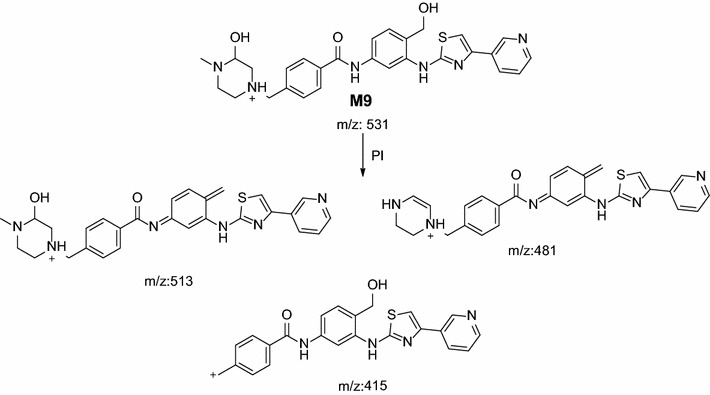
Supposed PIs of M9

In the case of M9, parent ion at *m/z* 531 was fragmented to ions at 513, 415, 381 and 123 (Fig. 11). Metabolic pathways for M9 were supposed to be benzyl hydroxylation and hydroxylation of methyl piperazine ring (Scheme 8).

**Fig. 12:**
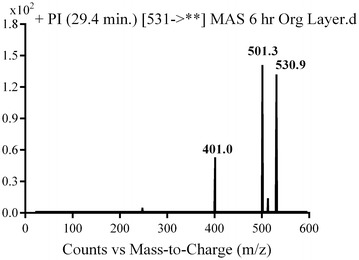
PI mass spectrum of parent ion (M10) at *m/z* 531

**Scheme 9:**
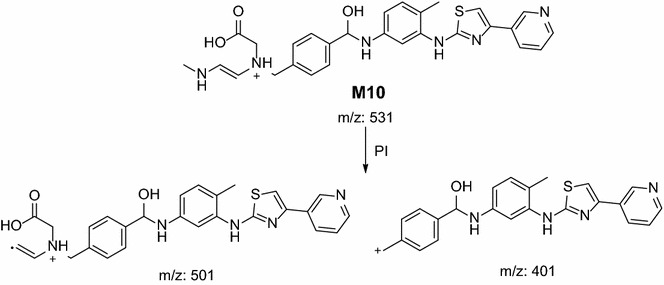
Supposed PIs of M10

In the case of M10, parent ion at *m/z* 531 was fragmented to ions at 501 and 401 (Fig. 12). Metabolic pathways for M10 were supposed to be oxidative cleavage of N-methyl piperazine ring to carboxylic acid (Scheme 9).

#### M11 in vivo phase I metabolite

M11 was detected at *m/z* 547 in mass scan spectrum of the urine organic extract. PI chromatogram of urine organic extract at *m/z* 547 showed one peak at 30.72 min. PI scan for M11 at *m/z* 547 gave daughter ions at *m/z* 511. Metabolic reactions for M11 metabolite were supposed to be hydroxylation of benzylic carbon, oxidation of pyridine nitrogen and oxidation of piperazine nitrogen.

#### In vivo phase I oxidative deamination metabolic pathway (MA1, MA2 and MA3)

The loss of the piperazine moiety by oxidative deamination and rapid further oxidation of the intermediate aldehyde to a carboxylic acid metabolite were observed for MA1, MA2 and MA3 in the aqueous layer of the urine/ACN mixture. Fragmentation of parent ions at *m/z* 431 and at *m/z* 447 gave different daughter ions. The structure of each metabolite was supposed.

**Fig. 13:**
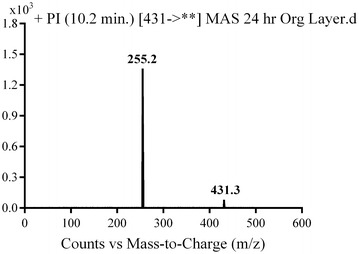
PI mass spectrum of parent ion (MA1) at *m/z* 431

**Scheme 10:**
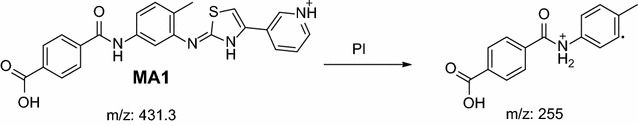
Supposed PIs of MA1

MA1 was detected at *m/z* 431 in mass scan spectrum of the aqueous layer urine extract. PI chromatogram of urine aqueous extract at *m/z* 431 showed one peak at 10.2 min. PI scan for MA1 at *m/z* 431 gave daughter ions at *m/z* 255 (Fig. 13). The daughter ion at *m/z* 255 supposed the loss of the piperazine moiety by oxidative deamination and rapid further oxidation of the intermediate aldehyde to a carboxylic acid (Scheme 10).

**Fig. 14:**
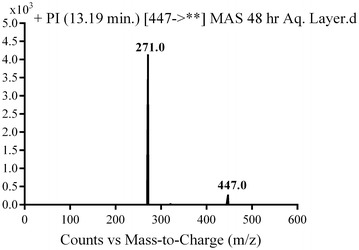
PI mass spectrum of parent ion (MA2) at *m/z* 447

**Fig. 15:**
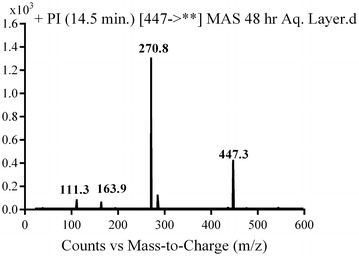
PI mass spectrum of parent ion (MA3) at *m/z* 447

MA2 and MA3 were detected at *m/z* 447 in mass scan spectrum of the aqueous layer urine extract. PI chromatogram of urine aqueous extract at *m/z* 447 showed two peaks at 18.6 and 19.5 min. PI scan for MA2 and MA3 at *m/z* 447 gave different daughter ions at two different retention times (Figs. 14 and 15).

**Scheme 11:**
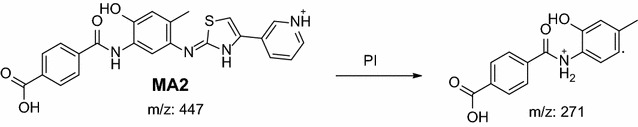
Supposed PIs of MA2

In the case of MA2, the daughter ion at *m/z* 271 supposed the loss of the piperazine moiety by oxidative deamination and rapid further oxidation of the intermediate aldehyde to a carboxylic acid in addition to phenyl hydroxylation (Scheme 11).

**Scheme 12:**
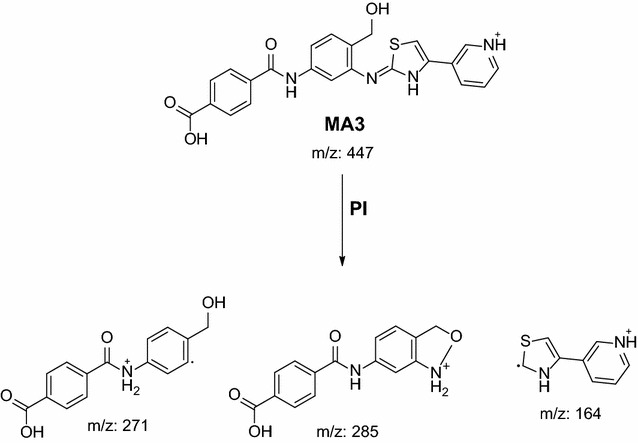
Supposed PIs of MA3

In the case of MA3, the daughter ion at *m/z* 271 supposed the loss of the piperazine moiety by oxidative deamination and rapid further oxidation of the intermediate aldehyde to a carboxylic acid. The other daughter ion at *m/z* 285 supposed benzyl hydroxylation (Scheme 12).

### Identification of in vivo phase II metabolic pathways of MST

Phase II metabolic pathways were supposed to be N-conjugation of MST and the N-demethyl metabolite with glucuronic acid, and glucuronidation of oxidative metabolites (Table 4). Phase II metabolites were found in the aqueous layer of the rat urine extract in a very small concentration compared to in vivo phase I metabolites. Excretion of all in vivo phase II metabolites in urine was observed after 12 h of rat dosing and disappeared rapidly after 48 h of rat dosing.

**Table 4:** In vivo phase II MST metabolites

	Mass scan	Daughter ions	Retention time (min)	Phase II metabolic pathway
MG1	675	499, 399	18.93	Direct N-conjugation with glucuronic acid
MG2	661	485	18.77	N-demethylation and direct N-conjugation with glucuronic acid
MG3	691	514.8	18.7	Glucuronidation of hydroxy MST at N-methyl piperazine ring
MG4	691	515.3, 414.9	19.46	Glucuronidation of hydroxy MST at benzyl carbon

#### MG1 in vivo phase II metabolite

**Fig. 16:**
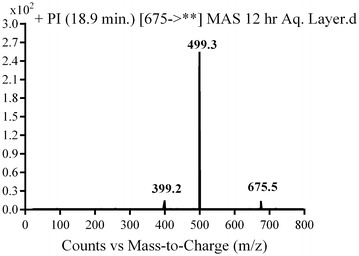
PI mass spectrum of parent ion (MG1) at *m/z* 675

**Scheme 13:**
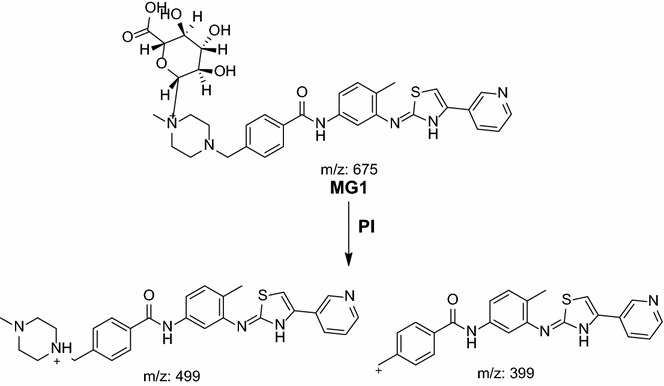
Supposed PIs of MG1

MG1 was detected at *m/z* 675 in mass scan spectrum of the aqueous layer urine extract. PI chromatogram of urine aqueous extract at *m/z* 675 showed one peak at 18.9 min. PI scan for MG1 at *m/z* 675 gave daughter ions at *m/z* 499 and 399 (Fig. 16). The daughter ion at *m/z* 399 supposed that direct N-conjugation of MST with glucuronic. The other daughter ion at 499 refers to the aglycone (MST) formed in the triple quadrupole by the loss of anhydroglucuronic acid (Scheme 13).

#### MG2 in vivo phase II metabolite

**Fig. 17:**
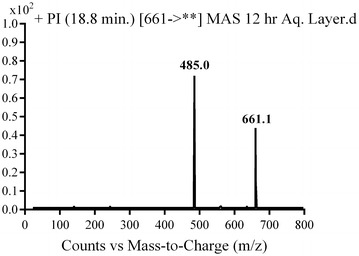
PI mass spectrum of parent ion (MG2) at *m/z* 661

**Scheme 14:**
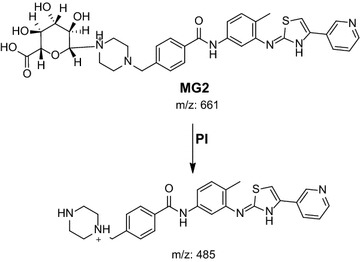
Supposed PIs of MG2

MG2 was detected at *m/z* 661 in mass scan spectrum of the aqueous layer urine extract. PI chromatogram of urine aqueous extract at *m/z* 661 showed one peak at 18.7 min. PI scan for MG2 at *m/z* 661 gave daughter ions at *m/z* 485 (Fig. 17). The daughter ion at 485 refers to the aglycone (N-demethyl MST) formed in the triple quadrupole by the loss of anhydroglucuronic acid (Scheme 14).

#### MG3 and MG4 in vivo Phase II metabolites

**Fig. 18:**
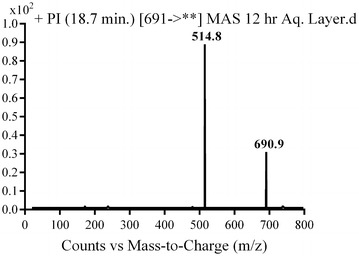
PI mass spectrum of parent ion (MG3) at *m/z* 691

**Fig. 19:**
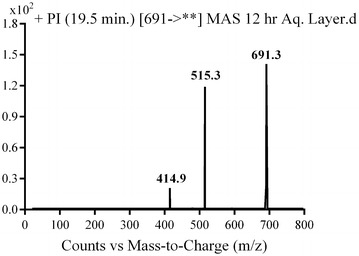
PI mass spectrum of parent ion (MG4) at *m/z* 691

MG3 and MG4 were detected at *m/z* 691 in mass scan spectrum of the aqueous layer urine extract. PI chromatogram of urine aqueous extract at *m/z* 691 showed two peaks at 18.6 and 19.5 min. PI scan for MG3 and MG4 at *m/z* 691 gave different daughter ions at two different retention times (Figs. 18, 19).

**Scheme 15:**
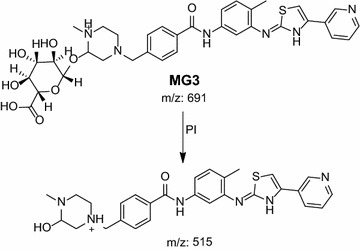
Supposed PIs of MG3

**Scheme 16:**
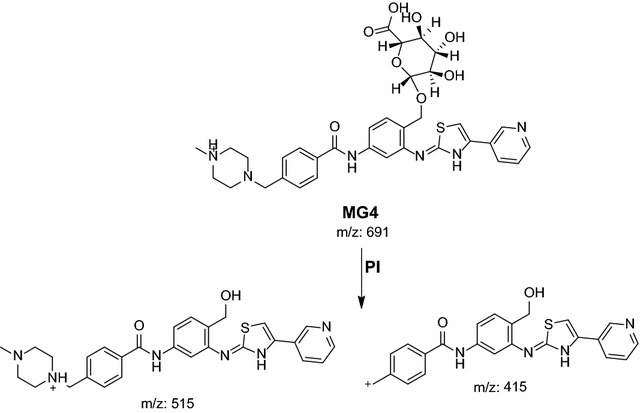
Supposed PIs of MG4

In the case of MG3, the daughter ion at *m/z* 515 supposed that direct O-glucuronidation of hydroxy MST. The daughter ion at 515 refers to the aglycone (hydroxy MST) formed in the triple quadrupole by the loss of anhydroglucuronic acid. (Scheme 15). Hydroxylation was supposed to be in the N-methyl piperazine ring. In the case of MG4, the daughter ion at *m/z* 515 supposed that direct O-glucuronidation of hydroxy MST. The daughter ion at 515 refers to the aglycone (hydroxy MST) formed in the triple quadrupole by the loss of anhydroglucuronic acid (Scheme 16). The other daughter at *m/z* 415 supposed that the hydroxylation of benzyl carbon.

## Conclusions

**Fig. 20:**
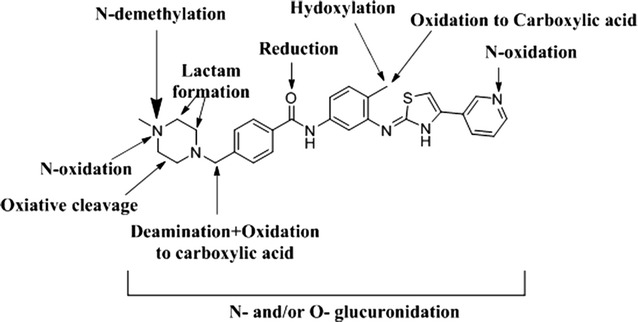
Chemical structure of MST and identified metabolic pathways in Rat. The main metabolic pathway was the N-demethylation

MST was excreted partially unchanged in rat urine. Twenty in vivo phase I metabolites were formed by oral dosing of MST to Sprague–Dawley rats through six metabolic pathways: N-demethylation, N-oxidation, oxidation, reduction, hydroxylation and oxidative deamination. Four in vivo phase II glucuronide conjugates were found in the aqueous layer of rat urine extract (Fig. 20).

## Additional file

**Additional file 1.** Additional figures.
