# Supplementary material for: Characterization of in vivo metabolites in rat urine following an oral dose of masitinib by liquid chromatography tandem mass spectrometry
Source: Chem Cent J. 2018 May 15;12:61. doi: 10.1186/s13065-018-0429-y (PMC5953916; doi:10.1186/s13065-018-0429-y)
Supplement: Supplementary file 1 — Additional file 1. Additional figures. [file 13065_2018_429_MOESM1_ESM.docx]

**Additional Figures**

**1-Rat urine control samples organic layer extract**

**
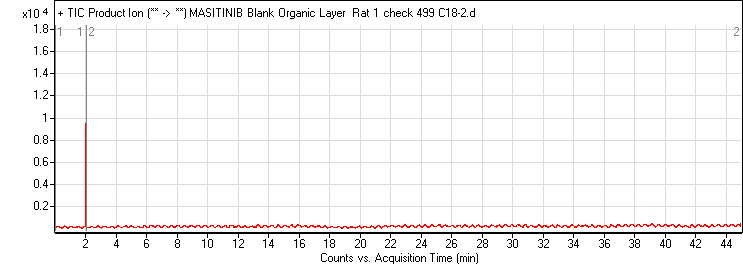
**

**Fig. S1.** PI chromatograms (red color) of molecular ions at *m/z* 499 of organic extract of control urine sample taken before masitinib dosing showing no peak at 24.9 min.

**
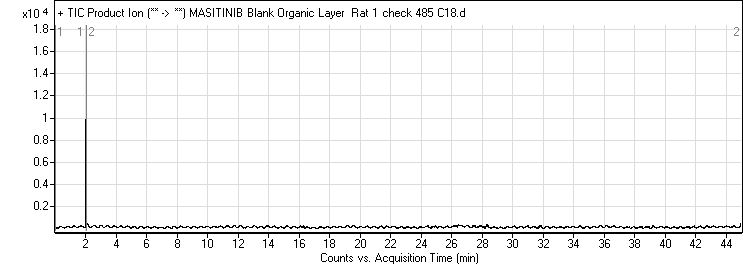
**

**Fig. S2.** PI chromatograms of molecular ions at *m/z* 485 of organic extract of control urine sample taken before masitinib dosing showing no peak at 27.9 min.

**
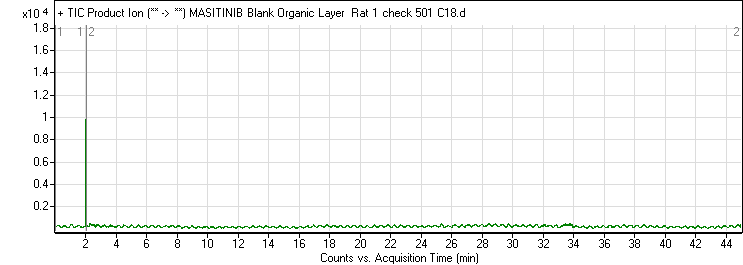
**

**Fig. S3.** PI chromatograms (green color) of molecular ions at *m/z* 501 of organic extract of control urine sample taken before masitinib dosing showing no peak at 24.4, 26.5 and 26.6 min.

**
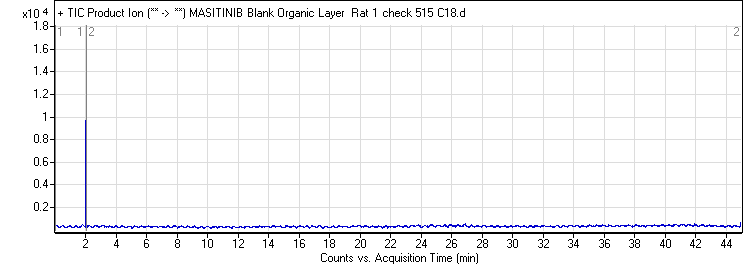
**

**Fig. S4.** PI chromatogram (blue color) of molecular ions at *m/z* 515 of organic extract of control urine sample taken before masitinib dosing showing no peak at 21.7, 22.2, 23, 23.1, 24 and 28 min.

**
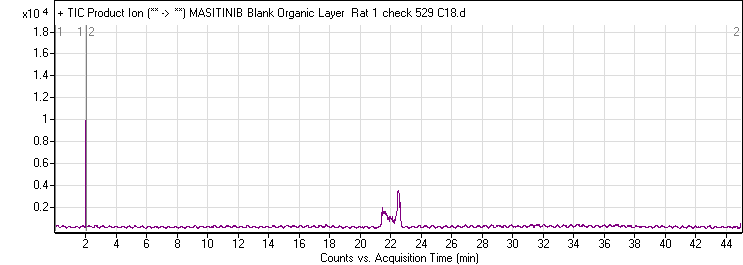
**

**Fig. S5.** PI chromatogram (purple color) of molecular ions at *m/z* 529 of organic extract of control urine sample taken before masitinib dosing showing no peak at 21.2 and 26.9 min.

**
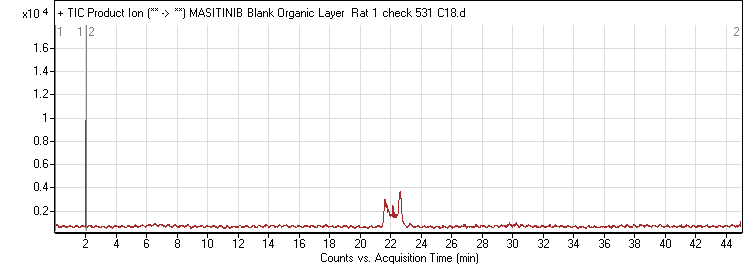
**

**Fig. S6.** PI chromatogram (red color) of molecular ions at *m/z* 531 of organic extract of control urine sample taken before masitinib dosing showing no peak at 26.7, 27.3 and 29.3 min.

**
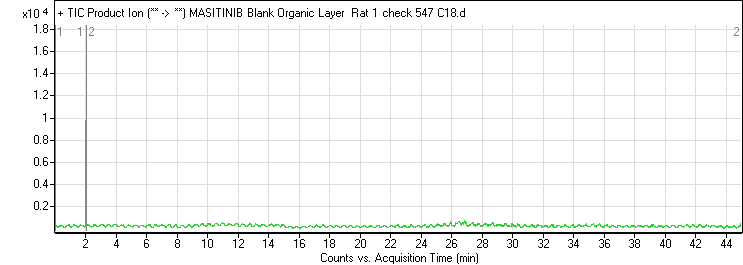
**

**Fig. S7.** PI chromatogram (green color) of molecular ions at *m/z* 547 of organic extract of control urine sample taken before masitinib dosing showing no peak at 30.7 min.

**2-Rat urine control samples aqueous layer extract**


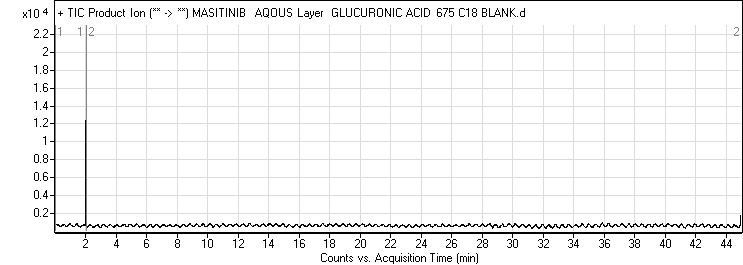


**Fig. S8.** PI chromatograms of molecular ions at *m/z* 675 of aqueous extract of control urine sample taken before masitinib dosing showing no peak at 18.9 min.


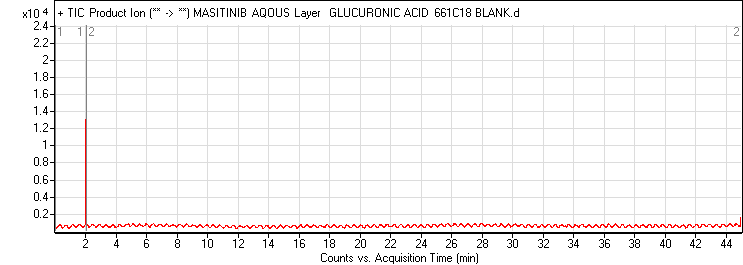


**Fig. S9.** PI chromatogram (red color) of molecular ions at *m/z* 661 of aqueous extract of control urine sample taken before masitinib dosing showing no peak at 18.77 min.


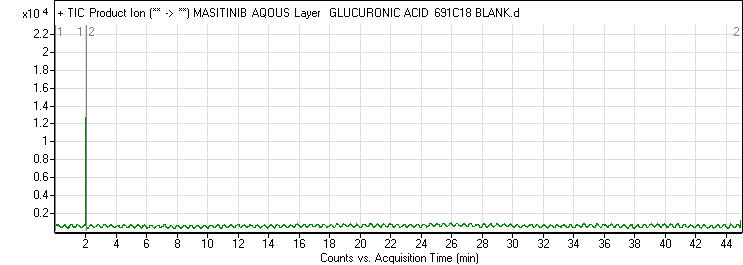


**Fig. S10.** PI chromatogram (green color) of molecular ions at *m/z* 691 of aqueous extract of control urine sample taken before masitinib dosing showing no peak at 18.77 and 19.46 min.

**3- EIC chromatograms for in vivo metabolites.**


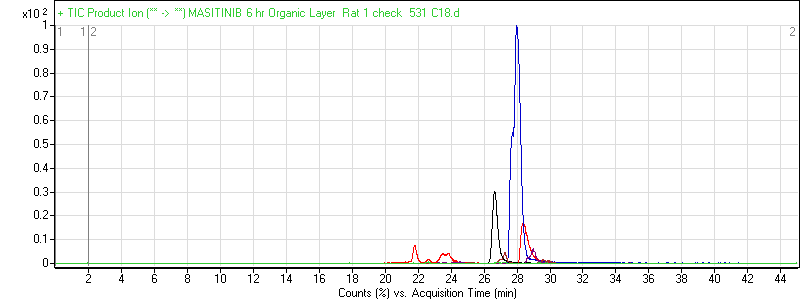


Masitinib

M1

**Fig. S11.** Overlayed PI chromatogram for masitinib and phase I metabolites indicating M1 is a major metabolite for masitinib.


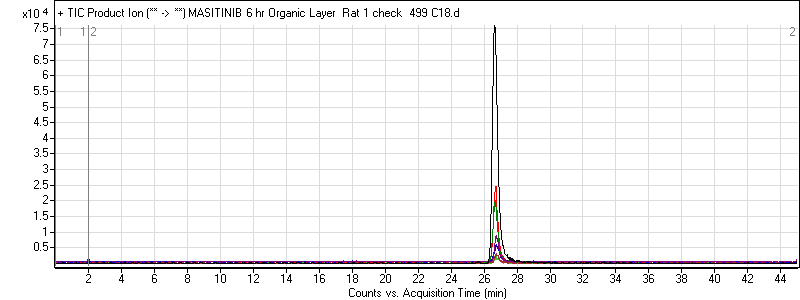


**Fig. S12.** Overlayed PI chromatograms of masitinib at *m/z* 499 (27 min.) at different collection times: 6 hr. (Black color), 12 hr. (red color), 18 hr. (green color), 24 hr. (pink color), 48 (blue color), 72 (orange) and 96 hr. (purple color).


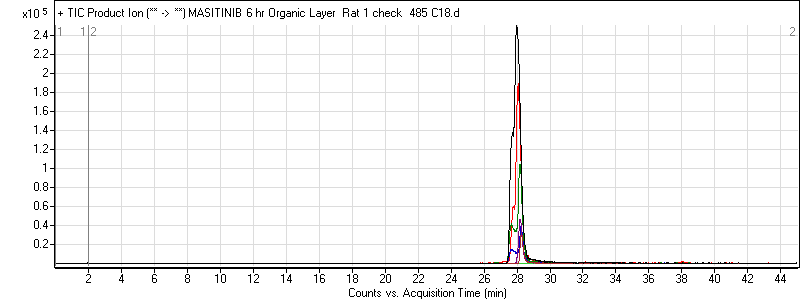


**Fig. S13.** Overlayed PI chromatograms of M1 at *m/z* 485 (27.9 min.) at different collection times: 6 hr. (Black color), 12 hr. (red color), 18 hr. (green color), 24 hr. (pink color), 48 (blue color), 72 (orange) and 96 hr. (purple color).


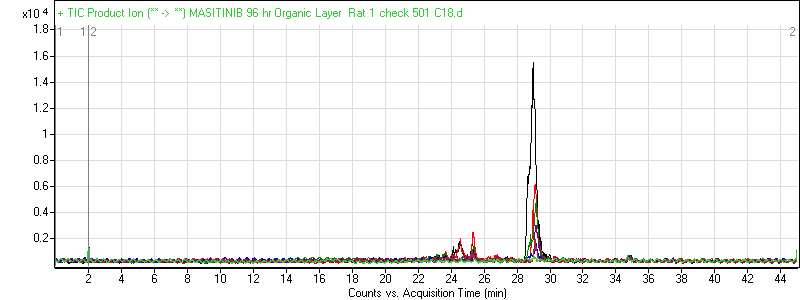


**Fig. S14.** Overlayed PI chromatograms of molecular ions at *m/z* 501 showing three peaks at 24.4, 26.5 and 28.9 min. for M3, M4 and M2, respectively at different collection times: 6 hr. (Black color), 12 hr. (red color), 18 hr. (green color), 24 hr. (pink color), 48 (blue color), 72 (orange) and 96 hr. (purple color).


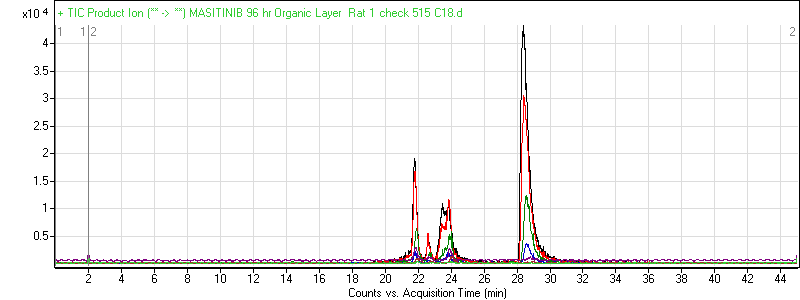


**Fig. S15.** Overlayed PI chromatograms of molecular ions at *m/z* 515 at specific time intervals showing six peaks at 21.7, 22.2, 23.0, 23.1, 24.0 and 28.0 for MO1, MO2, MO3, MO4, MO5 and MO6, respectively at different collection times: 6 hr. (Black color), 12 hr. (red color), 18 hr. (green color), 24 hr. (pink color), 48 (blue color), 72 (orange) and 96 hr. (purple color).


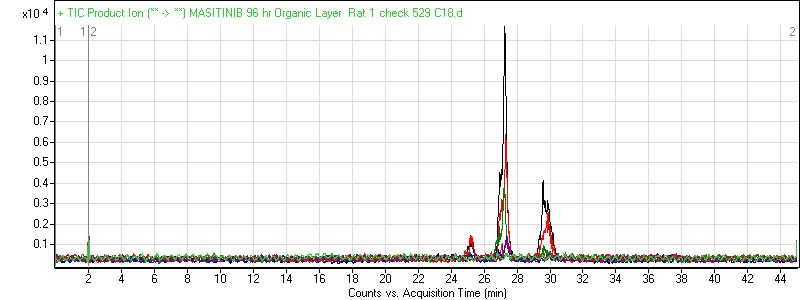


**Fig. S16.** Overlayed PI chromatograms of molecular ions at *m/z* 529 showing three peaks at 25.0, 26.9 and 29.6 min. for M5, M6 and M7, respectively at different collection times: 6 hr. (Black color), 12 hr. (red color), 18 hr. (green color), 24 hr. (pink color), 48 (blue color), 72 (orange) and 96 hr. (purple color).


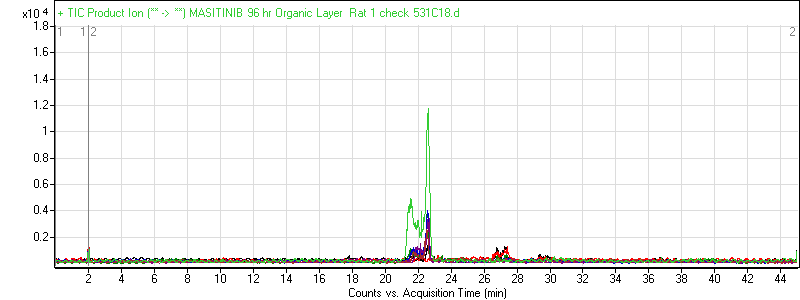


**Fig. S17.** Overlayed PI chromatograms of molecular ions at *m/z* 531 showing three peaks at 26.7, 27.3 and 29.3 min. for M8, M9 and M10, respectively at different collection times: 6 hr. (Black color), 12 hr. (red color), 18 hr. (green color), 24 hr. (pink color), 48 (blue color), 72 (orange) and 96 hr. (purple color).

***
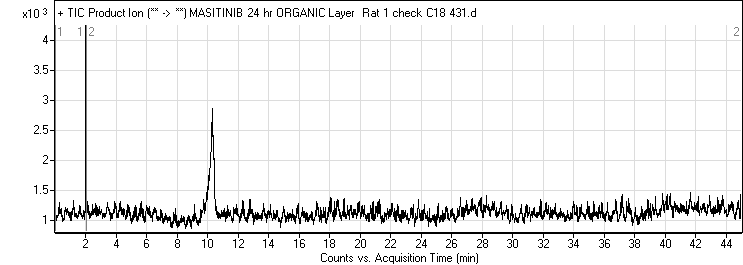
***

**Fig. S18.** PI chromatogram of molecular ion peak at *m/z* 431 showing MA1 peak at 10.2 min.


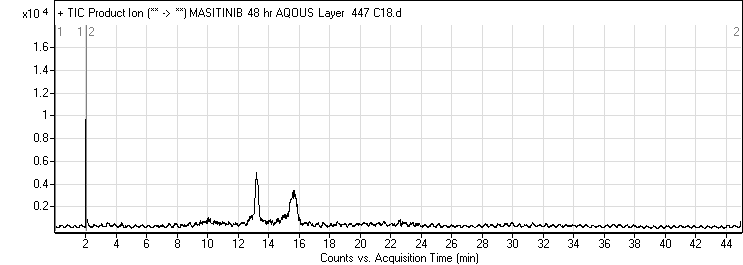


**Fig. S19.** PI chromatogram of molecular ion peak at *m/z* 447 showing MA2 and MA3 peaks at 13.2 and 14.5 min., respectively.


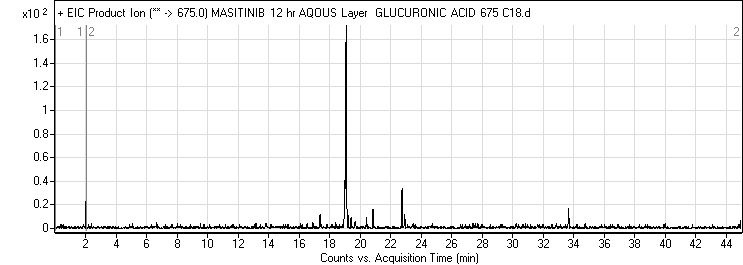


**Fig. S20.** PI chromatogram of molecular ion peak at *m/z* 675 showing MG1 peak at 18.9 min.


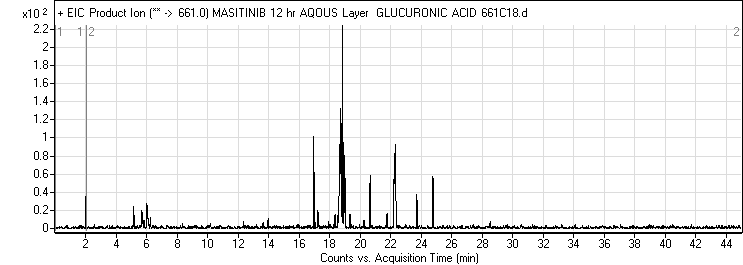


**Fig. S21.** PI chromatogram of molecular ion peak at *m/z* 661 showing MG2 peak at 18.7 min.


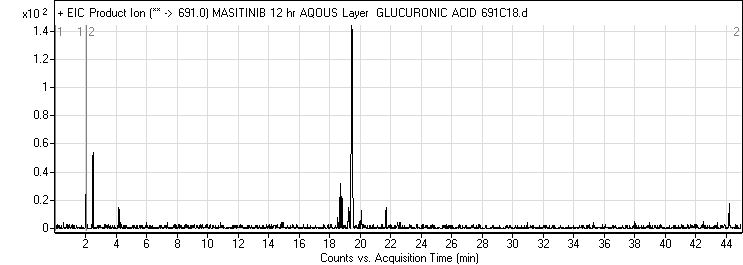


**Fig. S22.** PI chromatogram of molecular ion peak at *m/z* 691 showing MG3 peak at 18.6 min and MG4 peak at 19.5 min.

**4- Product ion mass spectra of in vivo metabolites that are reputed in in vitro metabolism of masitinib.**


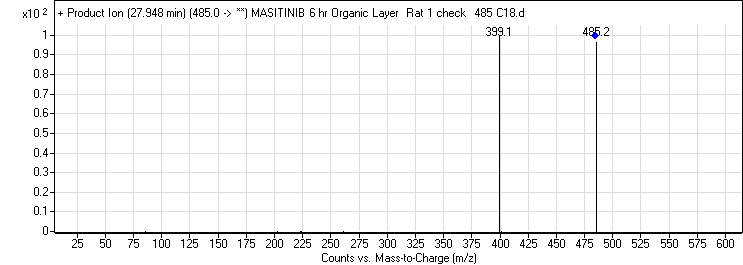


**Fig. S23.** PI mass spectrum of molecular ion peak (M1) at *m/z* 485.

***
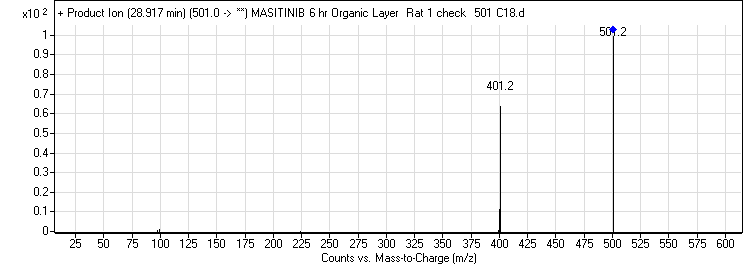
***

**Fig. S24.** PI mass spectrum of molecular ion peak (M2) at *m/z* 501.


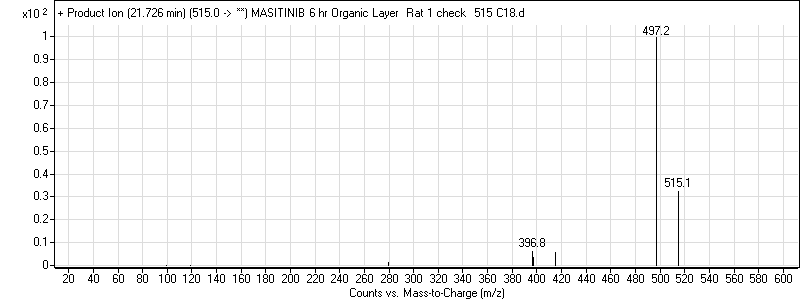


**Fig. S25.** PI mass spectrum of molecular ion peak (MO1) at *m/z* 515.


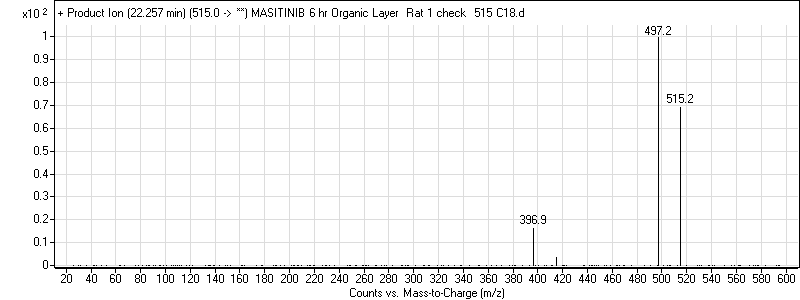


**Fig. S26.** PI mass spectrum of molecular ion peak (MO2) at *m/z* 515.


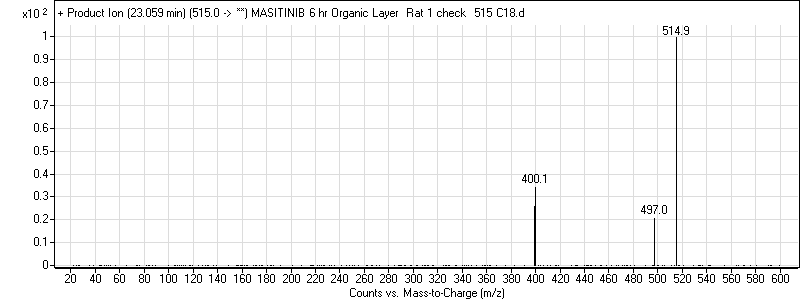


**Fig. S27.** PI mass spectrum of molecular ion peak (MO3) at *m/z* 515.


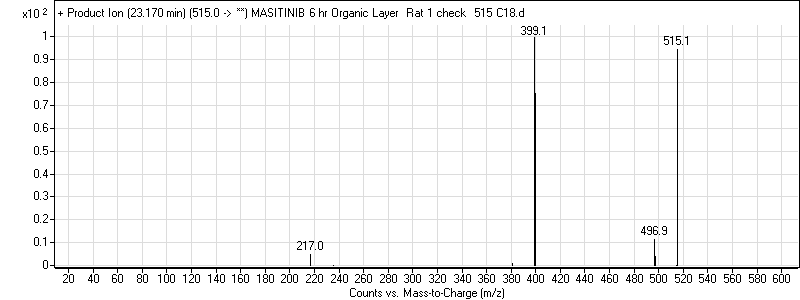


**Fig. S28.** PI mass spectrum of molecular ion peak (MO4) at *m/z* 515.


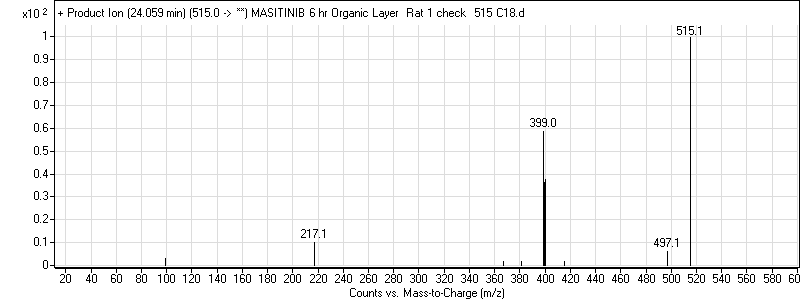


**Fig. S29.** PI mass spectrum of molecular ion peak (MO5) at *m/z* 515.


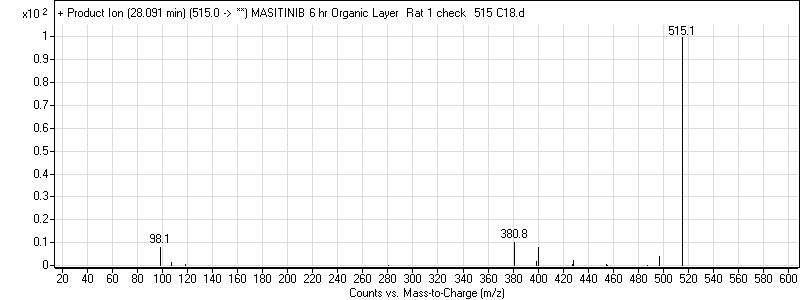


**Fig. S30.** PI mass spectrum of molecular ion peak (MO6) at *m/z* 515.


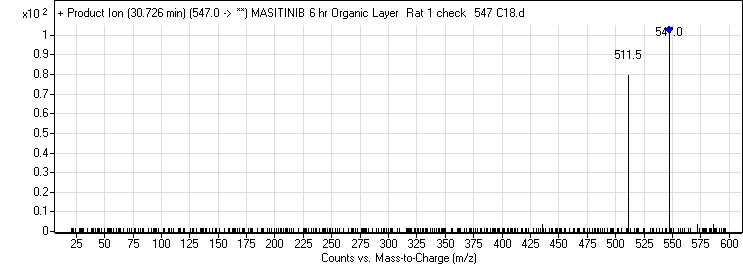


**Fig. S31.** PI mass spectrum of molecular ion peak (M11) at *m/z* 547.
